# Theta Coordinated Error-Driven Learning in the Hippocampus

**DOI:** 10.1371/journal.pcbi.1003067

**Published:** 2013-06-06

**Authors:** Nicholas Ketz, Srinimisha G. Morkonda, Randall C. O'Reilly

**Affiliations:** Department of Psychology, University of Colorado Boulder, Boulder, Colorado, United States of America; Indiana University, United States of America

## Abstract

The learning mechanism in the hippocampus has almost universally been assumed to be Hebbian in nature, where individual neurons in an engram join together with synaptic weight increases to support facilitated recall of memories later. However, it is also widely known that Hebbian learning mechanisms impose significant capacity constraints, and are generally less computationally powerful than learning mechanisms that take advantage of error signals. We show that the differential phase relationships of hippocampal subfields within the overall theta rhythm enable a powerful form of error-driven learning, which results in significantly greater capacity, as shown in computer simulations. In one phase of the theta cycle, the bidirectional connectivity between CA1 and entorhinal cortex can be trained in an error-driven fashion to learn to effectively encode the cortical inputs in a compact and sparse form over CA1. In a subsequent portion of the theta cycle, the system attempts to recall an existing memory, via the pathway from entorhinal cortex to CA3 and CA1. Finally the full theta cycle completes when a strong target encoding representation of the current input is imposed onto the CA1 via direct projections from entorhinal cortex. The difference between this target encoding and the attempted recall of the same representation on CA1 constitutes an error signal that can drive the learning of CA3 to CA1 synapses. This CA3 to CA1 pathway is critical for enabling full reinstatement of recalled hippocampal memories out in cortex. Taken together, these new learning dynamics enable a much more robust, high-capacity model of hippocampal learning than was available previously under the classical Hebbian model.

## Author Summary

We present a novel hippocampal model based on the oscillatory dynamics of the theta rhythm, which enables the network to learn much more efficiently than the Hebbian form of learning that is widely assumed in most models. Specifically, two pathways, Tri-Synaptic and Mono-Synaptic, alternate in strength during theta oscillations to provide an alternation of encoding vs. recall bias in area CA1. The difference between these two states and the unaltered cortical input representation creates an error signal, which can drive powerful error-driven learning in both Tri-Synaptic and Mono-Synaptic pathways. Furthermore, the presence of these alternating modes of network behavior (encoding vs. recall) provide an intriguing target for future work examining how prefrontal control mechanisms can manipulate the behavior of the hippocampus.

## Introduction

Over the past half century the hippocampus has provided fertile ground for the work of mechanistic computational models to inform empirical research. From the earliest investigations into Long Term Potentiation to the complex dynamics of place cells, models of hippocampal function have enabled a greater understanding of how learning and memory emerges from more basic neural mechanisms in this remarkable brain area. The paradigmatic theoretical model guiding this work is the Hebb-Marr framework [1]–[3], which features the core idea that Hebbian learning wires together neurons that are firing together as part of a memory or engram representation, e.g., in the central area CA3 of the hippocampus. With these connections strengthened, the ability to *pattern complete* a partial memory cue to a full representation of the original memory is enhanced. For this pattern completion within CA3 to actually drive full memory recall, it must trigger a chain reaction of pattern completion throughout the cortex — although central to most theoretical accounts, the critical role of the CA1 in this larger pattern completion process has not been as widely recognized. Specifically, learning between CA3 and CA1 neurons must take place at memory encoding, to enable the CA1 to then drive entorhinal cortex (EC), which then drives the higher-level association cortex areas that are bidirectionally interconnected with it. This plasticity at the CA3 to CA1 synapses indeed may be the most important factor for subsequent memory recall [4]. It is the nature of this plasticity, and the learning that takes place in the bidirectional connections between EC and CA1, that is the focus of this paper.

We argue that, by taking into account the phase differences of firing for these areas within the overall theta cycle of the hippocampus [5], [6], a powerful error-driven form of learning emerges, which can result in much higher storage capacity than the standard Hebbian learning mechanism. Furthermore, these phase dynamics within the EC – CA1 bidirectional connections enable the CA1 to very naturally learn to be a sparse, invertible auto-encoder of the EC inputs, which has long been an important but somewhat implausibly implemented feature of our computational models [7]–[10]. Thus, this new model, which we refer to as the *theta-phase* hippocampus model, in reference to the theta oscillation, provides a more unified and computationally powerful model of hippocampal function. This model also enables us to make more direct contact with a large base of evidence, in both humans and rodents, relating hippocampal EEG oscillations to learning and memory. Much of the progress within this literature has been made in animal electrophysiology targeting hippocampal representation during spatial navigation and recall, while evidence from human EEG and intracranial recordings of oscillatory interactions also shows connections to episodic memory.

Modeling work, originally developed within the spatial navigation literature, suggested that connectivity between hippocampal subregions is coordinated via the 3 to 8 Hz EEG theta oscillation [5], [6]. This work has also been extended into a more general framework of hippocampal function including a proposed extension from spatial navigation into episodic memory [8], [11]. These investigations provide the foundation for the theta-phase model described in the current work, in terms of establishing the existence and functional role of the oscillatory coordination of hippocampal subregions within an encoding and retrieval dynamic. We build upon this foundation by showing how these dynamics can lead to error-driven learning, and a concomitant increase in overall storage capacity for the system. The implementation of this theta-phase model is based directly on the Complimentary Learning Systems neural network model of the hippocampus [7], [9], [10], which is implemented within the Local, Error-driven, and Associative, Biologically Realistic Algorithm (Leabra) framework [12], [13]. We assess the impact of the theta-phase error-driven learning mechanisms by comparing it with an otherwise identical model that uses a Hebbian learning rule, while varying the number of units within the Dentate Gyrus (DG) and area CA3, and measuring the models' recall on a varying number of learned patterns. These learned patterns are presented at test with 25 percent of the pattern missing, and the models are compared on their ability to complete the missing portion of the pattern. Results show the error-driven signal performs significantly better than the Hebbian learning rule.

## Materials and Methods

### Hippocampal Architecture

**Figure 1:**
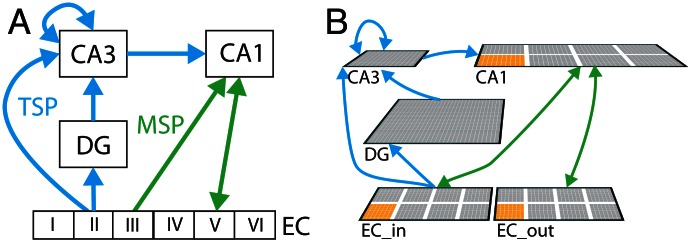
Hippocampal connectivity. *A)* Schematic of hippocampal connections with Entorhinal Cortex(EC) *B)* Image of neural network model used in this work on the right. Two pathways are highlighted: the Mono-Synaptic Pathway (MSP) in green, and the Tri-Synaptic Pathway (TSP) in blue. An individual EC *slot* is highlighted in orange within the neural network on the right.

The model used in the current work is built upon a series of structural and functional hypotheses based on anatomical and physiological data, which have been captured in the complementary learning systems (CLS) model of the hippocampus [7], [9]. The Entorhinal Cortex (EC) in the model is assumed to be the cortical gateway to the hippocampus. This gateway feeds through the *trisynaptic pathway* (TSP) to the Dentate Gyrus (DG), CA3, and then to CA1. Similarly, there is a parallel connection through the *monosynaptic pathway* (MSP) from the EC to the CA1 (and back) (Figure 1).

The TSP connections via the perforant path from EC to DG and CA3 are broadly diffuse, and support the conjunctive binding of various distributed pieces of information into an overall episodic memory representation in the CA3. The CA3 has sparse and highly separable patterns of activity (which are further pattern-separated via the very sparse DG layer), resulting in substantially reduced interference from synaptic weight changes, thus enabling rapid learning of novel episodic or conjunctive information [7]. To recall existing memories, the recurrent connections in CA3, along with plasticity in the EC to CA3, as well as the DG to CA3 connections, support pattern completion of missing information from retrieval cues.

For pattern completion in CA3 to have any effect on the rest of the brain, there must be a way to map the CA3 representation back out to the neocortex. This occurs via connections from CA3 to CA1 (the Schaffer collateral pathway), and then from CA1 back to EC, which then projects back out to the cortex to fill in the full memory representation in the cortical areas where it can actually be used in further cognitive processing. This Schaffer collateral pathway is a key focus of the theta-phase model, where we can train synapses in this pathway according to an error-driven learning signal, instead of the standard Hebbian signal assumed in other existing models.

The MSP between EC and CA1 is also essential for supporting memory retrieval, in a way that is often under-appreciated in the literature. This pathway is topologically organized, not diffuse, which we capture by organizing the simulated neurons in EC and CA1 into mutually interconnected *slots*, presumably encoding different separable elements across all the cortical areas that converge on the EC [14]. This slot architecture (Figure 1) enables the MSP to develop separable *invertible pathways* where a given EC input pattern can be encoded over a sparser representation in the corresponding CA1 slot, and this CA1 representation can in turn recover the full original EC slot pattern. The topographic nature of this CA1 representation is important for providing a mapping from cortex into the hippocampus and back out again. Weight adjustments along the TSP form conjunctive representations that bind information across the topography of EC and are important for recreating a previously experienced state from incomplete inputs (i.e., pattern completion). The Schaffer collaterals (the connection between CA3 and CA1) provide the translation between these two types of representations, allowing the conjunctive representations learned in the TSP to influence the topographic representations within CA1, and subsequently back out to EC. In our previous CLS models, we have trained these topographic slot mapping weights between EC and CA1 in an offline manner prior to training the full hippocampal network. The new theta-phase learning mechanisms now enables us to train this important MSP pathway in a very natural manner, at the same time as the rest of the hippocampal system learns.

To summarize, after learning, the model recollects studied items by reactivating the original patterns via the trained weighted connections between areas. The accuracy of this recall is scored as a simple comparison between the originally studied 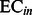 pattern and the recollected 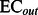 pattern. If the input pattern corresponds to a non-studied pattern, or even if individual components of the pattern were previously studied, but not together, the conjunctive nature of the CA3 representations will minimize the extent to which recall occurs. Conversely, when previously studied patterns are presented in an incomplete or noisy input format, these weights allow the hippocampus to recall the originally studied pattern.

### Theta Phase Learning

As noted previously, the original Complementary Learning Systems (CLS) hippocampal model pretrained the invertible mapping between EC and CA1 on a vocabulary of possible patterns for a single slot [9]. The resulting weights for the connections within this individual slot network were then replicated across all EC–CA1 slots (see Figure 1 where an individual slot is highlighted) in the MSP. This restricts the space of inputs possible to the vocabulary of patterns in which the slot network was trained.

The alternative approach adopted in this work utilizes simultaneous, independent learning along both the MSP and the TSP. This dual-pathway learning is motivated by physiological recordings within the subfields of rat hippocampi, along with mathematical models of hippocampal function [6], in terms of the 3–8 Hz oscillatory EEG signal known as theta. The theta oscillation can be found throughout the hippocampus and surrounding cortex, however it is strongest and most consistent when recorded within the region separating CA1 and DG known as the hippocampal fissure. For this reason all references to theta oscillations will be referring to the EEG signal measured at the hippocampal fissure.

**Figure 2:**
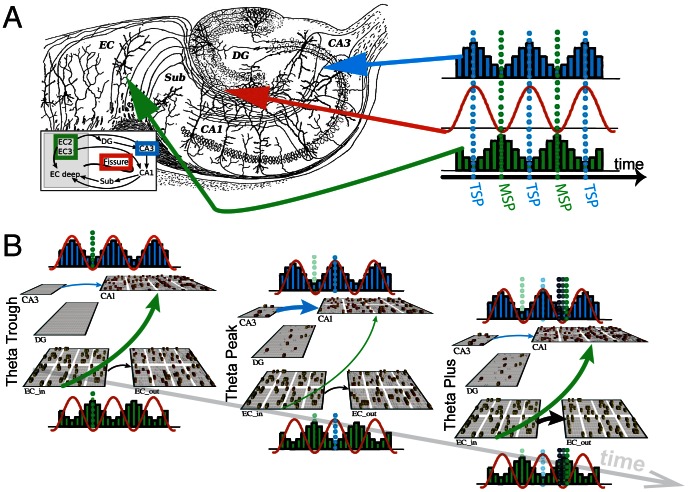
Diagram of relation to physiological data and computational model. Figure adapted from [6] *A)* Relation of current-source/sink analysis within subfields of the hippocampus and the fissure recorded theta oscillation. The blue histogram shows the strength of the Tri-Synaptic Pathway's(TSP) influence on area CA1 over time, and the green histogram shows the Mono-Synaptic Pathway's(MSP) influence on CA1 on the same time line. The orange line represents the fissure recorded theta oscillation in reference to these histograms. Dotted lines show the points of maximum influence from either the TSP or MSP on CA1. *B)* Visual depiction of computational model shown at three sequential time points, with arrow weight highlighting the manipulated connection strengths at those time points; connections not depicted imply there was no modification of connection strength. The three time points of interest, theta peak, theta trough, and theta plus are shown with the influence of the MSP(shown in green) on CA1 strong at theta's trough, the influence of the TSP(shown in blue) on CA1 strong at the peak, and the influence of 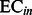 on 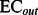 (shown in black), as well as 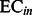 to CA1 strong during theta plus. The transition from theta plus to the following theta trough is shown in the far right network.

Figure 2A shows an illustration of hippocampal subfield dynamics in relation to the fissure recorded theta oscillation shown in red. This cartoon, derived from current source density analysis [6], [15], shows the current sinks into area CA1 alternatively originating from either area CA3 in blue or EC layers II and III in green. At the trough of fissure recorded theta, EC sources into CA1 are at their peak and area CA3 is at its minimum. This implies that EC has a strong influence over synaptic potentials within area CA1 at this time. At the peak of fissure recorded theta, CA3 sources are at their peak and EC influence has diminished. This again suggests that CA3 input to area CA1 is now the dominant influence, and EC is less so as compared to the trough of the theta oscillation.

These dynamics are modeled within the neural network as, for any given input pattern, three distinct time points of activation: Theta Trough (TT), Theta Peak (TP), and Theta Plus (+) as shown in Figure 2B. These three time points are modeled as three independent settling processes across simulated neurons within the differential equation described in eq. (1). The patterns of activation that arise from these three time points are used to train the weighted connections along the MSP and the TSP, where the equations for the error-driven weight changes at these synapses are shown in eqs. (6) and (7).

Specifically, input patterns are projected onto 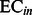 which is then allowed to project to CA1 and subsequently to area 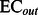, while CA3 input to CA1 is inhibited. This creates a pattern of activation dominated by the MSP which is then used to drive learning within these connections. This is denoted as superscript *^TT^* in eq. (6) for “Theta Trough”, as this time point is analogous to the connectivity dynamics at the trough of theta oscillations, where EC strongly influences CA1, and CA3 influence is relatively low. Following this, CA3 input onto CA1 is released from inhibition while the influence from 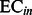 onto CA1 is diminished. This corresponds to the Theta Peak (denoted as *^TP^* in eq. (7)); a time point that reflects strong influence from CA3 onto CA1. This time point is analogous to the peak of the fissure recorded theta oscillation where EC input to CA1 is weak, while CA3 input is strong.

The final *plus* stage of activation (denoted with the ^+^ in eqs. (6) and (7)) corresponds to 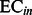 projecting onto 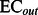 and area CA1, and 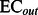 projecting back onto CA1. The representations within 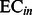 and 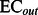 will remain relatively static due to the direct connection between them, which then forces CA1 to settle into a representation that respects this symmetric mapping between 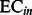 and 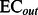. This provides the veridical *ground truth* in the error-driven learning signal. In reference to eq. (3), this pattern of activation is used for the plus stage learning signal in contrast to the MSP's *^TT^* and the TSP's *^TP^* minus stage.

The alteration of these connections' strength are manipulated in the model by simply denying information flow through specific subregion projections at select points in the settling process of the differential equation shown in eq. (1). The three particular projections that are manipulated in the model are 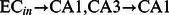, and 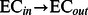 where the pattern of manipulation that these projections are subjected to are highlighted in Table 1. All other connections within the network have no error-driven component to their weight adjustments, only Hebbian, as seen in eq. (8).

**Table 1:** Table of connection strength between subfields as a function of theta phase.

Phase	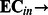 CA1	CA3 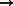 CA1	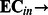 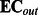
Theta Trough	+	−	−
Theta Peak	−	+	−
Theta Plus	+	−	+

The ‘+’ symbols represent unaltered connections, and ‘−’ symbols represent a fully inhibited connection.

### Model Validation

The validation process adopted in this work is to compare the theta-phase learning model described above with a simple Hebbian learning model. The critical connections that utilize an error-driven learning signal within the theta-phase model are the Mono-Synaptic Pathway (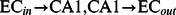), as well as the Schaffer collaterals (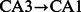). In contrast, these connections in the comparison model use a purely Hebbian learning rule. The task run across both models is a simple capacity test such that each model is trained for 15 repetitions of an input pattern set (referred to as 15 *epochs*), and the performance of the two models is then tested by measuring the accuracy of the recalled patterns of activation given an input cue which has 25 percent of the trained pattern missing.

We explored three training regimes to contrast error-driven vs. Hebbian learning. First, both the MSP and TSP utilized an error-driven learning signal and was compared to a full Hebbian network. We then compared the contribution of these two pathways by using error-driven learning within either the MSP and not the TSP, or conversely within the TSP and not the MSP. Finally, to better compare against earlier models where the MSP pathway was pretrained in advance, we compared pretrained vs. non-pretrained MSP. In the pretrained MSP, only the MSP pathway was trained for 15 epochs (on the same patterns used for the overall training), followed by integrated training of both TSP and MSP as described above. In the non-pretrained MSP, both pathways were trained in the integrated fashion from the start.

The question of how network performance scales is addressed by varying the training set size, and network size across multiple levels of these two variables. The size of the input pattern set is varied from 40 to 800 patterns to get a measure of model performance across small and large training sets, with the assumption that better performance on larger training sets is more reflective of hippocampal function. Similarly, the size of the network itself was varied by increasing the number of units within the CA3 and DG layers, while holding a constant ratio between them. This is done to try and maintain a connection to the original biological constraints of the hippocampal circuit, and for this reason a ratio of 5 DG units to 1 CA3 unit was adopted, as this generally reflects the ratio in the human hippocampus [14],[16]. Maintaining this ratio, the total units within CA3 were varied from 10 to 100 units, which in turn corresponds to a varying of DG units from 50 to 500.

Finally, input patterns were constructed, and memory retrieval performance measured, based on the slot topology in the EC layers (as highlighted in Figure 1). This slot structure is intended to capture the modality segregation within EC, and within each slot we assume there is a *vocabulary* of different patterns, which reflect the representational repertoire within those modalities. We generated a vocabulary of 100 distributed activity patterns, with a minimum hamming distance of 10 between each vocabulary pattern generated. A complete input pattern used in the model validation process was then constructed by selecting a single pattern from these 100 vocabulary patterns for each of the EC slots. With 8 slots, a total of 100^8^ (n*_patterns_* raised to the n*_slots_* power) unique 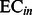 patterns are possible, however only 800 were used in the testing of these models. These vocabulary patterns were similarly used to estimate error within the networks' output by comparing, within a given slot, the output pattern of activation with all other vocabulary patterns. If, for the given input pattern, the slots' output at the 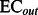 layer is closest to the vocabulary pattern it was trained on, it is considered correct, and otherwise considered incorrect. This closest-pattern calculation is done across each of the slots for every input pattern, and if any slot shows an incorrect response the network output for that input pattern is counted as incorrect. This measurement is referred to as *Name Error* in the results section, and is thought to better represent the potential for clean up of hippocampal output as compared to more standard measures such as Sum Squared Error (SSE). It also has the advantage of not requiring any further threshold or other parameterization. It should be noted that this measure of error, compared to a SSE, deemphasizes single unit based errors in output in favor of an emphasis on distributed patterns of error across groups of units.

### Learning Framework

The model is implemented in the Leabra framework which uses a combination of supervised and Hebbian learning [13]. What follows is a coarse description of the essential components within this framework necessary for understanding the current work. The activation function for a given unit is a threshold based neuronal model with continuous valued spike rate as output. Each neuron's membrane potential (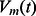) is updated using the following differential equation:
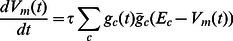
(1)Here, 3 channels (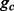) summed across in the membrane potential calculation are: 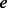 excitatory input, 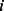 inhibitory input, and 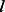 leak current. Excitatory input is calculated as the average over all weighted inputs coming into a unit (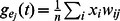), where 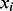 is the activity of sending unit 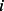 and 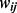 is the weighted connection between sending unit 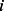 and receiving unit 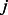. All principal weights between units are excitatory while local circuit inhibition controls positive feedback loops. Leabra assumes a winner take all dynamic through a set-point inhibitory current (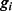), producing a *kWTA* (k-Winners-Take-All) dynamic. kWTA is computed via a uniform level of inhibitory current for all units within a layer. Finally leak current (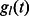) is a constant value set to 0.1

Activation of communication (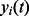 for a given unit 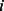) with other units is a thresholded function of membrane potential:
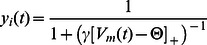
(2)Here 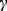 is the gain factor which is set to a constant value of 100, and 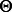 is the firing threshold value which is set to a constant of 0.5 within a units dynamic range of 0 to 1.

The Leabra framework utilizes a biologically plausible error-driven learning algorithm which is equivalent to Contrastive Hebbian Learning (CHL) [17]. Leabra uses two stages of activation; the 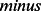 stage is the initial activation or expected output of the network, while the 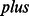 stage is the provided target output activation. The Leabra weight updating component between sending units (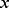) and receiving units (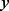) is thus calculated as:
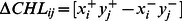
(3)The ^+^ and ^−^ superscripts represent the plus and minus phase components respectively. In addition to the error-driven learning of CHL, a pure form of Hebbian learning is also used. Here the weight change is calculated using only the target, or plus phase, activations
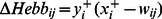
(4)This learning rule can be seen as computing the expected value of the sending unit's activity conditional on the receiver's activity [13]. Finally these two learning rules are proportionally weighted (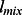) along with a learning rate parameter, 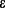, for the combined learning rule used in this work:
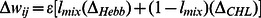
(5)


The theta-phase learning approach uses the learning framework described above within a particular dual-pathway architecture. The target, or *plus*, component of the error signal (superscript ‘+’ in eqs. (6) and (7)) is activation acquired from the 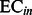 layer projected onto 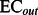, and allowed to propagate back on to area CA1 which settles into a pattern of activation constrained by static representations in 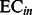 and 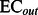. Similarly 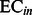 projects along the TSP providing a plus phase activation within DG and CA3, however projections from CA3 onto CA1 are inhibited. Error signals used in weight adjustment are then calculated by taking the difference between this plus phase activation and the two distinct time points within the theta cycle (peak and trough), yielding two distinct error signals. Specifically, the MSP connections are adjusted according to an error signal acquired from the difference between plus phase activation and activation patterns acquired during the trough of theta (superscript TT in eq. (6)). It is critical to remember in the trough of theta there is no influence on 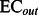 representations from area 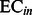, while in the plus phase 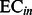 projects onto 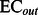. The difference in CA1 activation patterns at the conclusions of these two phases allows for the calculation of an error signal that is used to adjust the weighted connections within the MSP. Similarly, the TSP connections are adjusted according to an error signal acquired from the difference between the plus phase activation and the activation during the peak of theta (superscript TP in eq. (7)). In the peak of theta CA3 has a strong influence on CA1, while in the plus phase CA1 is influenced solely by the MSP. This change in CA1 representations allows for a error signal tailored to best adjust the TSP connections to more closely match the stimulus driven representation of the plus phase activations. All other connections, within the network, i.e. 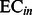 to DG and CA3, DG to CA3, and recurrent connections within CA3, have no error-driven component to their weight adjustment (eq. (8)).
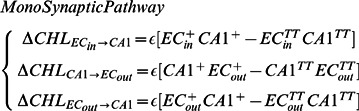
(6)

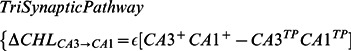
(7)

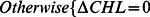
(8)


Settling dynamics within the network are dictated by the temporal evolution of Equation 1. This dynamic process, within every unit, is allowed 30 time steps to settle into its equilibrium state for each of the three phases within the theta cycle, thus yielding a total of 90 time steps for each full theta oscillation. All activation values within the network are reset to 0 at the onset of theta trough but are allowed to be carried over from trough to peak and finally from peak to the plus phase without alteration. All manipulations of Hebbian vs. error-driven learning where done via the *lmix* parameter as shown in eq. (5). Values used to instantiate full Hebbian learning implies a lmix value of 1, while error-driven learning used a lmix value 0f 0.001. This implies that error-driven networks also used a very small amount of Hebbian weight adjustment which we believe is implicit in normal neural circuitry.

## Results/Discussion

**Figure 3:**
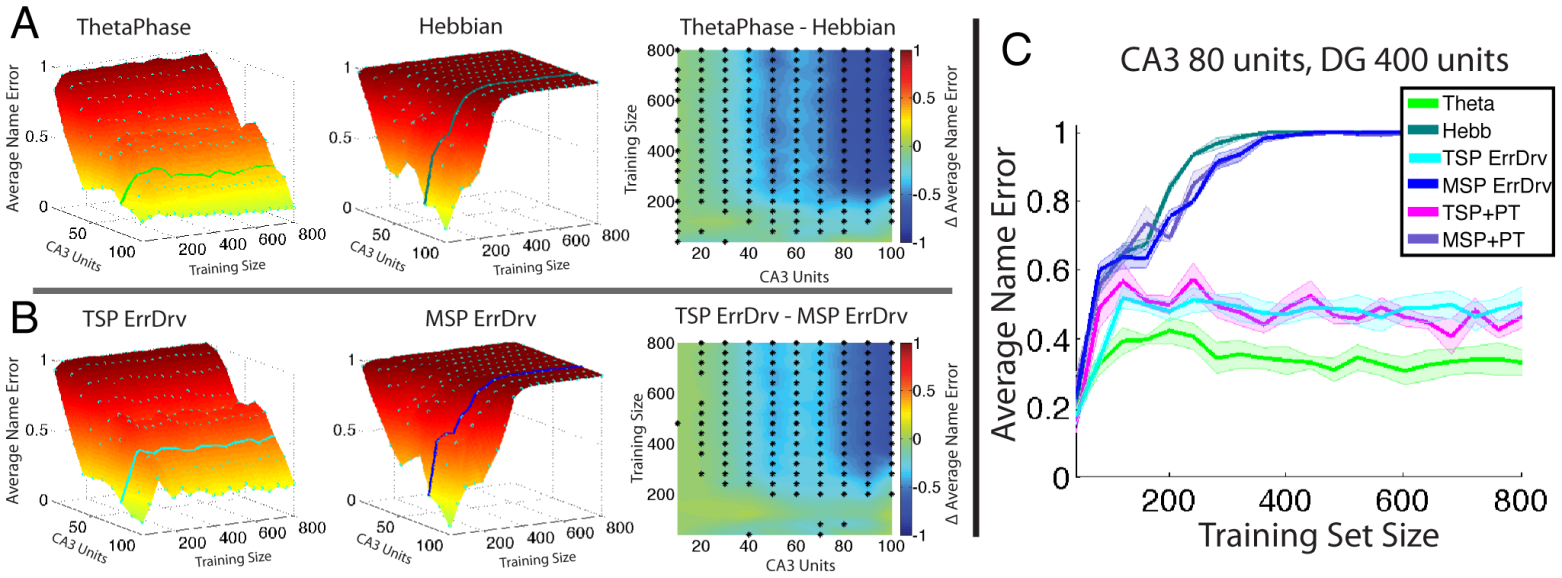
Comparison of network performance contrasting Hebbian and Error Driven learning rules. Network performance plotted across various network sizes and training set sizes. *A)* Surface plots of the average Name Error across the full training set plotted on the left for both the theta-phase (i.e. error-driven learning in both MSP and TSP) and the Hebbian network, and the difference between theta-phase and Hebbian surfaces plotted on the right with an asterisk showing values significantly(p<0.005) different from 0. Cyan dots in the surface plots are data points where performance was measured *B)* Same Name Error surface plots with the left panel (labeled TSP ErrDrv) showing performance from a network with error-driven learning in the TSP and Hebbian learning the MSP. Middle panel (labeled MSP ErrDrv) shows performance from a network with Hebbian learning in the TSP and error-driven learning in the MSP. Difference between these two shown on the right. *C)* Plot of network performance taken from A and B for a single network size of 80 CA3 units and 400 DG units. Line color is shown in A and B where these data were extracted from the surface plots, and the magenta and purple lines(labeled TSP+PT and MSP+PT respectively) come from networks with the same error-driven configuration as in B, however these networks were pretrained on the input patters for 15 epochs within the MSP. Full surface plots for these two networks are not shown.

Figure 3 shows the comparison of various network configurations. In panel A the theta-phase network with error-driven learning in the MSP and TSP is compared with a fully Hebbian learning network across various network sizes and trained input pattern set sizes. Plots are shown as a function of network size, where the number of CA3 units are shown on the x-axis which implies that the number of DG units for that network are 5 times that of CA3. Training set size, shown on the y-axis refers to the number of patterns a given network was trained and tested on. Surface plots of the average Name Error, on the z-axis, across the full training set are shown on the left for both the theta-phase and the Hebbian network. Each cyan dot in the surface plots represents a measured data point where both network types were tested in the network-size by training-set-size space. Each data point is the average within network type across 5 random weight initializations. These points were then fit to a 3D surface for visualization. The difference between theta-phase and Hebbian surfaces is shown on the right. These differences are compared using a random bootstrap method where Name Error values are sampled with replacement from both network types into two groups and a distribution of difference values is calculated to produce a null hypothesis. Data points with p values less than 0.005 are shown in the difference plot with an asterisk.

Similarly, in panel B of Figure 3 a more fine grained follow up test of performance shows a network with error-driven learning in the TSP and Hebbian learning in the MSP (labeled TSP ErrDrv) compared against a network with Hebbian learning in the TSP and error-driven learning in the MSP. This secondary test attempts to evaluate the relative importance of error-driven learning within these two pathways on overall performance. These results are then further tested by comparing pretrained MSP connections to non-pretrained MSP connections. These results are not shown in a similar style as Figures 3A and B as these pretrained networks yielded results nearly identical to non-pretrained networks. Figure 3C shows performance plots from individual networks within these three comparisons; here the overlap in pretrained and non-pretrained results can be seen in comparison to the other two comparisons shown in Figures 3A and B.

Results, shown in panel A of Figure 3, of the comparison between network models shows that the error-driven learning, provided by the theta coordination of subfield influence on CA1, out-performs the purely Hebbian based learning network. Investigating this relationship further, in panel B of Figure 3 it is shown that the crucial connection that leads to this benefit is between CA3 and CA1 along the TSP. There is little difference in performance when the TSP uses Hebbian learning (plot labeled MSP ErrDrv) compared to when the full network is exclusively using Hebbian learning. Conversely, when the TSP (specifically the connection between CA3 and CA1) takes advantage of the error-driven learning signal, performance is dramatically increased, and approaches Name Error levels achieved when both TSP and MSP are using error-driven learning signals (shown in Figure 3B and C). Contrasting performance from the full ThetaPhase network with the TSP error-driven network shows that there is indeed some performance benefit in the ThetaPhase network compared to TSP error-driven network, suggesting some synergy between the TSP and MSP over and above the benefit from the TSP error-driven network alone.

Our comparison of the effects of pretraining on the MSP, as was done in our earlier models, revealed very little difference as shown in Figure 3C. This is of considerable practical benefit, as it is often difficult to anticipate the full range of input pattern variability needed for pretraining, and it also increases the overall plausibility of our model, by eliminating any need for this extra step in the model.

These results provide insight as to how these learning signals compare across multiple network sizes and varying training set sizes. Looking at the difference in network performance we can see a divergence towards better error-driven performance as training size and network size increases. Many hippocampal models used within the literature test on relatively small training sets and with small network sizes; usually of the size required for the task or phenomena being modeled. Results from the current work suggest that Hebbian performance may not scale with these dimensions as expected, and that a more robust learning signal such as that provided by error-driven learning may be necessary to provide realistic performance in more ecologically valid network sizes and training set sizes. Given the significant performance advantages of the error-driven learning mechanism, and its biological support in the theta-phase coordination process, it would be surprising if the biological hippocampus did not also leverage this form of error-driven learning. In sum, we argue that this model represents a significant advantage over the existing Hebbian-based models of hippocampal learning, and can provide a predictive framework for future empirical studies.

The idea of temporal differentiation between Mono-Synaptic and the Tri-Synaptic pathways along the theta wave, as shown in previous hippocampal modeling work [5], [6], provides a well founded framework for how theta oscillations interact with behavior. The key contribution of this work to these models is a demonstration that the invertible mapping in and out of area CA1 along the Mono-Synaptic Pathway can be learned in tandem with the connections along the Tri-Synaptic Pathway, and that these oscillatory dynamics enable a form of powerful error-driven learning. Further, these results suggest that error-driven learning in the Schaffer collaterals connecting CA3 to CA1 are a crucial component in stabilizing this invertible mapping in the Mono-Synaptic Pathway, and providing the performance advantage shown in Figure 3.

The mapping of distributed representations into and out of area CA1 is a problem that has not been adequately addressed in previous models. Many models have used a simplified symmetric representation between hippocampal subregions [5], [6]. This allows for a transparent interpretation of subregion processing, however it reduces the ecological validity of the model's processing. An early model of episodic memory allowed for learning within this invertible mapping between EC and CA1, however the representations used were relatively small and simplified [8]. The current work shows that error-driven learning is a key component behind the requirement of relatively complex representational transformation between subregions. The attempt to match the hippocampal architecture and representational complexity within this work provides insight into these more subtle issues that are often assumed in other models of the hippocampus. The simulations done in this work show that the representational transformation into and out of the hippocampus is a non-negligible problem, and that more robust learning signals than the standard Hebbian model are required for accurate recall within large training data and small network sizes.

The current model provides a simplified version of oscillatory processes within a discretized time frame, as compared to previous models [5], [6]. The peak and trough time points being modeled in the current work can be thought of as stimulus driven at the trough of theta, and recall driven at the peak of theta [6], however these processes are implemented within the model as two relatively discontinuous patterns of activation that get integrated together when calculating the weight changes in the learning algorithm. Additionally, the plus phase of activation, i.e. the ground truth within the error calculation, is proposed as a projection of the superficial EC layers onto the deep layers of the EC. Computationally within the model this is implemented after both the trough and peak of the theta oscillation have completed, however we conceptualize the theta cycle to begin on the trough of the oscillation where the MSP is strongly active, and we therefore speculate that this plus phase projection would occur within the descending theta cycle following the peak but just before the trough. In Figure 2 we show the plus phase to occur at the trough of theta, however the model predicts that the plus phase would occur anytime between theta peak and theta trough. In some sense the plus phase is a transition from theta peak to theta trough where the onset of the plus phase is marked by the inhibition of the TSP and a projection from the superficial layers of EC to the deep layers. This allows for the error-driven contrasting of this plus phase pattern of activation with the preceding theta trough and theta peak patterns. Indeed, laminar recordings from Entorhinal Cortex support this theta phase reversal in deep layers compared to superficial [18], and a recent investigation into the microcircuits within EC layers supports the increased firing from superficial EC to deep EC just preceding the trough of ongoing theta oscillations [19]. Future electrophysiological work could test these temporal dynamics further by stimulating at these various stages of the theta wave to try and disrupt or enhance this theoretical cascade of activation.

Previous models have labeled activation patterns associated with theta peak and trough as *Encoding* during the trough, and *Retrieval* during the peak, which our model also captures [5], [6], [20]. This separation of functionality between the two pathways might allow for other systems to interact with the nominal theta cycle to influence these processes and thereby bias the hippocampus towards one process over the other. A growing base of empirical evidence within the rodent literature suggests that oscillatory coherence within the theta band between frontal regions and the hippocampus is correlated with successful retrieval [21]–[23]. In humans these interactions could provide the framework for some form of volitional control over either encoding or retrieval. Future empirical work in humans could probe this relationship between encoding and retrieval within the hippocampus as well as its interaction with other systems. The current model would suggest that disruption of the theta oscillation during the trough of theta would alter the encoding of new experiences, while disruption at the peak of theta would alter the retrieval of previous experiences.

The question of how incoming stimuli align to these phase dynamics is somewhat unclear, however constraints from previous empirical work do exist. There is evidence suggesting that theta oscillations show a phase resetting approximately 200 ms after stimulus onset [24]–[26]. The entry point into the theta wave on these phase resets, however, show a difference in study vs. test items where test items enter on the descending wave of theta while study items enter on the ascending wave. Our model suggests that there would be a plus phase following the descending theta wave, and would be evident through the projection from the superficial layers of EC to the deep layers. This task dependent phase reset could help to target this plus phase dynamic, and potentially determine whether it is more associated with start of a given theta oscillation or with the end.

There are many limitations within the current work in regards to the scope of biological components, and we do not mean to suggest that this model accurately reflects all aspects of hippocampal function. For example, the discrete nature of the two time points modeled, i.e. trough and peak, within the theta cycle could be better approximated by having a continuous change of activation after the plus phase. The current work simplifies the more continuous change of activation at the end of a Theta cycle by resetting activation after the plus phase. Additionally, there are hippocampal subfields, in particular the Subiculum [27], which are not included within this model. We are currently exploring the addition of a Subiculum layer within our model which modulates the learning rate of connections into CA1. The Subiculum-mediated modulation focuses on increasing the learning rate for novel stimuli, and reducing the learning rate for well learned Tri-Synaptic Pathway (TSP) representations, theoretically allowing for the reduction of interference in the otherwise purely Hebbian learning in the TSP (e.g., in perforant pathway projections from EC to CA3). Although no current explorations are underway, area CA2 could also provide an augmentation to our model of the MSP [28]. This area would fit in as a intermediary between the 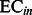 and CA1, providing a non-topographic representation across the slots of Entorhinal Cortex, and potentially increasing the learning capacity along this pathway.

In conclusion, within the subfields modeled, we have accurately represented the known connectivity and topology using a biologically motivated neural network framework. Further, we have included coordination between those subfields through the currently understood inhibitory processes as modulated by theta oscillations. Building upon this framework in future projects can provide a strong foundation in the known biological constraints, and representational complexity of the hippocampal circuit.
